# Neuroimaging Correlates of Treatment Response to Transcranial Magnetic Stimulation in Bipolar Depression: A Systematic Review

**DOI:** 10.3390/brainsci13050801

**Published:** 2023-05-15

**Authors:** Ahmad Shamabadi, Hanie Karimi, Giulia Cattarinussi, Hossein Sanjari Moghaddam, Shahin Akhondzadeh, Fabio Sambataro, Giandomenico Schiena, Giuseppe Delvecchio

**Affiliations:** 1 Psychiatric Research Center, Roozbeh Psychiatric Hospital, Tehran University of Medical Sciences, Tehran M9HV+R6Q, Irang.h.sanjarimoghaddam@gmail.com (H.S.M.); s.akhond@neda.net (S.A.); 2 School of Medicine, Tehran University of Medical Sciences, Tehran P94V+8MF, Iran; 3 Department of Neuroscience (DNS), Padua Neuroscience Center, University of Padova, 35131 Padua, Italy; giulia.cattarinussi@unipd.it (G.C.); fabio.sambataro@unipd.it (F.S.); 4 Department of Neurosciences and Mental Health, Fondazione IRCCS Ca’ Granda Ospedale Maggiore Policlinico, 20122 Milan, Italy

**Keywords:** bipolar depression, brain imaging, functional neuroimaging, systematic review, transcranial magnetic stimulations

## Abstract

Transcranial magnetic stimulation (TMS) has become a promising strategy for bipolar disorder (BD). This study reviews neuroimaging findings, indicating functional, structural, and metabolic brain changes associated with TMS in BD. Web of Science, Embase, Medline, and Google Scholar were searched without any restrictions for studies investigating neuroimaging biomarkers, through structural magnetic resonance imaging (MRI), diffusion tensor imaging (DTI), functional MRI (fMRI), magnetic resonance spectroscopy (MRS), positron emission tomography (PET), and single photon emission computed tomography (SPECT), in association with response to TMS in patients with BD. Eleven studies were included (fMRI = 4, MRI = 1, PET = 3, SPECT = 2, and MRS = 1). Important fMRI predictors of response to repetitive TMS (rTMS) included higher connectivity of emotion regulation and executive control regions. Prominent MRI predictors included lower ventromedial prefrontal cortex connectivity and lower superior frontal and caudal middle frontal volumes. SPECT studies found hypoconnectivity of the uncus/parahippocampal cortex and right thalamus in non-responders. The post-rTMS changes using fMRI mostly showed increased connectivity among the areas neighboring the coil. Increased blood perfusion was reported post-rTMS in PET and SPECT studies. Treatment response comparison between unipolar depression and BD revealed almost equal responses. Neuroimaging evidence suggests various correlates of response to rTMS in BD, which needs to be further replicated in future studies.

## 1. Introduction

Bipolar disorder (BD), a severe mental disorder characterized by severe mood fluctuations and altered cognitive processes, continues to be one of the leading causes of global disability [1]. Depressive symptoms are present in 70% and 80% of the symptomatic phases of BD type 1 and 2, respectively, making up the main burden of this disorder. BD is associated with increased mortality and morbidity, including high suicide risk and co-occurring medical diseases such as diabetes, cardiovascular diseases, stroke, and metabolic syndrome [2]. Similar genetic susceptibility traits and neurotransmitter activities of unipolar and bipolar disorders and higher depression risk in the relatives of individuals with mania and high comorbidity of mania and depression had convinced researchers that these two disorders are not distinct and are two poles of the same disorder [3]. However, more recently this notion has been challenged, considering the differences in clinical and epidemiological characteristics between unipolar and bipolar disorders, including less anxiety and agitation and more mood dysregulation, younger onset age, more rapid recurrence, and more frequent mood swings in BD [3]. Diagnosis is not easy and is usually delayed. Indeed, BD is often recognized as unipolar depression for 6–8 years until the mood switch to hypomania or mania occurs [2]. The average age of onset is reported to be 12–24 years in BD type I, which is higher in BD type II and the highest is seen in unipolar depression; however, these findings might vary in different cultures and countries [4].

The first-line treatment approaches for BD include pharmacotherapy and psychotherapy [1]. The most prominent and well-known treatment guidelines are as follows: Canadian Network for Mood and Anxiety Treatments (CANMAT), American Psychiatric Association (APA), National Institute for Clinical Excellence (NICE), Maudsley guidelines and the Canadian Psychiatric Association treatment guidelines; the treatment strategies somehow differ among the mentioned guidelines, however, recently, some efforts have been made to apply a unified guideline. For instance, the treatment of mixed episodes of BD is the same in all guidelines, indicating the first-line treatment with Valproate followed by Quetiapine or Olanzapine. In contrast, the treatment approach used for a depressive episode of BD is somehow different in the mentioned guidelines although the unified guideline recommends utilizing Lithium either alone or combined with serotonin reuptake inhibitors (SSRIs); which will be followed by lamotrigine or quetiapine or olanzapine–fluoxetine combination [5]. However, few treatments are proven to be consistently effective in acute episodes of BD, and there is even less evidence supporting the long-term efficacy of medications in preventing recurrences [2]. In recent years, since some alternative treatments, such as neurostimulation techniques, including repetitive transcranial magnetic stimulation (rTMS), deep brain stimulation (DBS), and electroconvulsive therapy (ECT) had satisfactory outcomes in unipolar depression, they have been also proposed for the treatment of BD [6,7,8,9]. 

Among the above-mentioned techniques, rTMS has become a promising and effective strategy for managing BD, due to its safety profile, non-invasive nature, and improved focality, with response rates of approximately 40–50% [1,10]. rTMS is operated through an electromagnetic coil, which modulates the electrical activity of the brain by stimulating the cerebral cortex coupled with the induction of electrical currents [11]. Notably, it has been reported that active rTMS is more effective than sham rTMS in improving clinical outcomes in bipolar depression [6,12,13]. In addition to the clinical outcome, the response to rTMS treatment has also been assessed through neuroimaging parameter changes. Indeed, the alterations in the activity of single neural regions or different brain networks, often observed in BD patients compared to healthy subjects [14,15] might also occur post-rTMS and could therefore be used as predictors of outcomes [16]. 

To increase the effect of rTMS in the treatment of BD, the identification of response predictors, either clinical or biological, might be useful. Understandably, the cost and time needed for this treatment have prompted researchers to investigate these predictors further [17]. It has also been shown that the rTMS predictive accuracy of neuroimaging markers is higher than demographic or clinical ones [18].

Therefore, in recent years, researchers have explored the neural correlates of response to rTMS employing different neuroimaging techniques, including functional, structural, and metabolic imaging. In particular, functional connectivity alterations among different cortical regions using blood oxygen level-dependent (BOLD) signal with functional magnetic resonance imaging (fMRI) have been reported [19], in addition to structural abnormalities evaluated with structural magnetic resonance imaging (MRI) [20] and diffusion-weighted MRI [21]. Furthermore, investigations examining brain metabolic’ changes with magnetic resonance spectroscopy (MRS) [22], cerebral blood flow using positron emission tomography (PET) [23], and single-photon emission computerized tomography (SPECT) [24] have also been conducted in individuals with bipolar depression after rTMS.

Considering the lack of a study systematically examining the neuroimaging outcomes of rTMS in BD and investigating neuroimaging predictors of response in this population, this study aims to collect and provide an overview of all available evidence on neuroimaging findings, indicating functional, structural, and metabolic brain changes associated with rTMS in individuals with BD.

## 2. Materials and Methods

The Preferred Reporting Items for Systematic Reviews and Meta-Analyses (PRISMA) statement [25] was considered in the design of this systematic review. The study protocol was published in the International Prospective Register of Systematic Reviews (PROSPERO), code CRD42022375039.

### 2.1. Searching

To obtain the data, A.S. searched ISI Web of Science Core Collection, Embase, and Medline databases on 31 July 2022, without any limitations on timespan, language, and document type. Table A1, Appendix A presents the terms and fields searched in each database. Searching Google Scholar and reviewing the references of included studies were additional sources.

### 2.2. Inclusion Process

A.S. and H.K. independently screened and assessed the studies. If needed, G.C.’s opinion was obtained. Based on the PICO criteria, the following criteria were necessary for inclusion: (i) diagnosis of active BD; (ii) TMS intervention without any restriction to the protocol used; and (iii) evaluation of outcomes using a neuroimaging modality, including structural MRI, diffusion tensor imaging (DTI), fMRI, MRS, PET, and SPECT. There was no requirement for a control group, and no limitation was considered in the setting. Editorials, case reports, and review studies were excluded. In addition, articles not published in peer-reviewed journals and pre-clinical studies, including in vitro and animal ones, were excluded.

**Figure 1:**
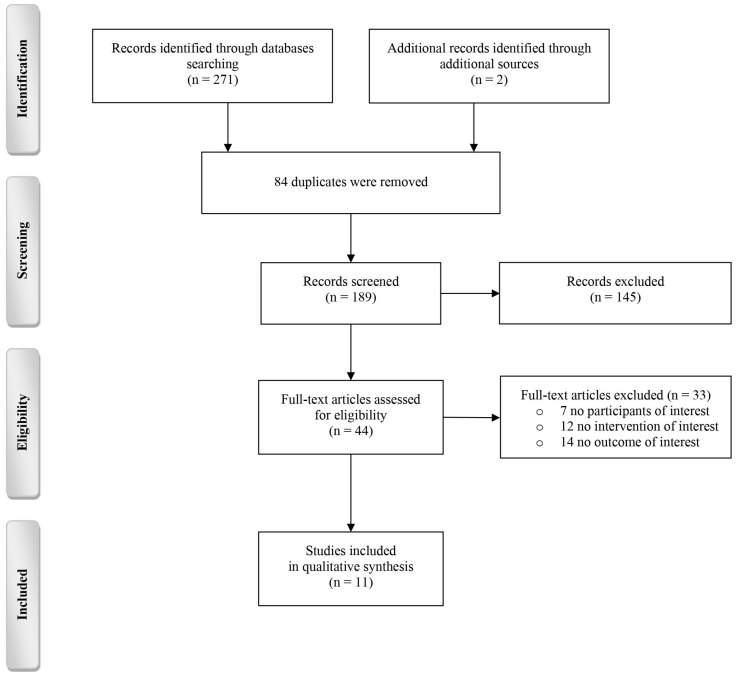
Processing the selection of studies.

Figure 1 summarizes the search and inclusion process of the studies, and the number of results obtained from each database is given in Table A1, Appendix A. The search yielded 273 articles, 189 of which remained after removing duplicates. After the title/abstract screening, 145 citations did not meet the inclusion criteria. After full-text reading, 33 other articles were excluded, thus resulting in 11 eligible studies. The reasons for excluding studies whose full texts were assessed for eligibility are given in Supplementary Materials.

### 2.3. Data Extraction

H.K. and A.S. extracted the following data from each included study in parallel and consulted with G.C. in case of disagreement: study type and design, number of participants, intervention characteristics, imaging modality, studied brain networks, number of treatment responders, and baseline and post-treatment findings and correlations. The primary outcome was the neuroimaging findings following the use of TMS in patients with BD. Instead, the correlation of response to TMS with other factors, the comparison of neuroimaging correlates of response to TMS in unipolar and bipolar depression, and adverse events following TMS in patients with BD were secondary outcomes. If necessary, the authors of the included studies were contacted.

## 3. Results

A total of 11 studies were included [1,16,17,19,22,23,24,26,27,28,29]. Of these, four employed resting-state fMRI, three PET, two SPECT, one MRS, and one MRI. In all studies, BD patients were in the depressive phase; no studies using manic BD patients were retrieved. The characteristics of the studies are listed in Table 1.

In the following sections, the neuroimaging findings post rTMS, as well as the baseline predictors of better treatment outcomes through different imaging techniques, will be presented for fMRI, MRI, PET, SPECT, and MRS studies.

### 3.1. Changes Relative to Baseline

#### 3.1.1. fMRI Investigations

With regards to fMRI studies, Li et al., (2004) [27], conducted one Hz prefrontal rTMS in six patients with unipolar depression and eight with bipolar depression and found increased activation in areas neighboring the coil in the prefrontal cortex, bilateral thalamus, putamen, parietal lobes and insula, ipsilateral hippocampus, right lateral orbitofrontal cortex, and left middle temporal gyrus (MTG). Moreover, significant deactivation was evident in the right ventromedial prefrontal cortex (VMPFC). It was also demonstrated that subcortical and prefrontal circuit (encompassing the bilateral putamen, bilateral frontal cortex, left mediodorsal and anterior nucleus of the thalamus, left insula, left hippocampus, right orbitofrontal cortex, and left pulvinar) functions were altered post-rTMS, along with the VMPFC deactivation, which is known to have a role in antidepressant response.

Furthermore, another study using the same modality by Salomons et al., (2014) [16], on 25 patients, 21 with unipolar depression and 4 with BD in depressive phase, indicated that improvement in Hamilton depression rating scale (HAM-D) scores were correlated with increased resting state functional connectivity (rs-FC) between the thalamus and the dorsomedial prefrontal cortex (DMPFC), as well as the decrease in the connectivity among DMPFC and insula, DMPFC and parahippocampal gyrus/amygdala, as well as between the subgenual region of the anterior cingulate cortex (sgACC) and caudate and sgACC and midcingulate cortex.

Finally, an investigation of rs-FC across 8 resting-state networks using fMRI was conducted by Godfrey et al., (2022) [19] on 26 depressed patients (24 with unipolar depression, 2 with bipolar depression). The authors found that lower salience network (SN) connectivity in prefrontal regions positively correlated with Montgomery-Åsberg Depression Rating Scale (MADRS) scores. Evaluation of the internetwork connectivity of the resting state networks showed higher post-rTMS rs-FC between posterior default mode network (pDMN) and SN, without, though, being related to antidepressant response. In addition to rs-FC, local spontaneous activity was examined by fractional amplitude of low-frequency fluctuations (fALFF), demonstrating an increase in fALFF in SN, pDMN, right frontoparietal (rFPN), left frontoparietal (lFPN), and central executive network (CEN). Notably, changes in fALFF values did not correlate with MADRS scores.

#### 3.1.2. Structural MRI Investigations

One structural MRI study carried out a whole-brain analysis to investigate the correlation between cortical thickness and volume with rTMS response in twenty-four patients with bipolar depression. The authors found that the lower volume of the left SFG and left caudal middle frontal gyrus (MFG) was associated with the rTMS response. However, global cortical thickness, left dorsolateral prefrontal cortex (DLPFC) thickness, and the cortical target distance from the scalp did not show significant differences across responders and non-responders [1].

#### 3.1.3. PET Investigations

Regarding the PET findings, a double-blind, placebo-controlled study was conducted on nine depressed BD patients using rTMS over the left prefrontal cortex at two frequencies of one Hz and 20 Hz using 15 O water PET (a technique used to facilitate the visualization and quantification of blood flow). It displayed an increased regional cerebral blood flow (rCBF) after conducting 20 Hz rTMS in the cingulate gyrus, prefrontal cortex, left amygdala, uncus, basal ganglia, bilateral insula, hippocampus, parahippocampus, cerebellum, and thalamus. One Hz rTMS resulted in decreased rCBF in small areas of the left medial temporal cortex, right prefrontal cortex, left amygdala, and left basal ganglia. Moreover, the authors found that global CBF (gCBF) increased after 20 Hz rTMS, but no change was seen in gCBF after one Hz rTMS. Regarding clinical changes, the authors observed that when the HAM-D scores improved with one of the frequencies (either one Hz or 20 Hz), the other frequency (20 Hz after 1 Hz or 1 Hz after 20 Hz) was associated with higher scores [23]. Nine years later, in 2009, Speer et al. conducted another study with 20 Hz, one Hz, and sham rTMS across BD and unipolar subjects and further reported the improvement of HAM-D score with either 1 or 20 Hz frequency and its deterioration with the other frequency, using FDG-PET/H152O PET [28].

#### 3.1.4. SPECT Investigations

A SPECT trial conducted on seven BD patients in the depressive phase investigated the effect of rTMS on brain regions by comparing the effects of active rTMS (with two different frequencies of 5 Hz and 20 Hz) and the placebo stimulation. The authors found a significant improvement in the active stimulation group, with no difference in fast or slow stimulation. However, rCBF changes were different after fast versus slow stimulation: the 20 Hz rTMS increased the rCBF in the left DLPFC and decreased it in the left hippocampus and left midcingulate compared with 5 Hz stimulation. Active rTMS was related to increased rCBF in the left MFG and right medial frontal lobe, besides a decreased activity across the left insula, left cingulate, and left uncus compared to baseline. The active rTMS group showed post-treatment lower activity in the bilateral parietal cortex and left thalamus compared to the placebo group. Comparing the alterations from baseline between active and placebo groups also showed that the left MTG had lower activity compared to baseline in the active rTMS group relative to the placebo group [29].

#### 3.1.5. MRS Investigations

A sham-controlled iTBS study coupled with a GABA-edited MRS was performed by Diederichs et al. [22]. The study was conducted on seventeen subjects with acute bipolar depression to examine whether bipolar depression had the same alterations in GABA levels as in two previously reported outcomes in unipolar depression [30,31]. Eighteen participants with bipolar depression (six with BD-1 and 12 with BD-2) were randomized into two active (n = 11) and sham (n = 7) iTBS groups. At baseline, there were similar GABA levels in the two groups, whereas after treatment, a significant GABA level rise in the medial prefrontal cortex (mPFC) region among active-iTBS group subjects was evident; notably, GABA levels were not correlated with the antidepressant effect or anhedonia.

### 3.2. Predictors

#### 3.2.1. fMRI Investigations

fMRI has been used to predict the rTMS response in BD patients in various studies including an open-label trial on four BD patients in the depressive phase, which found that higher baseline rs-FC between the DMPFC/sgCC and DLPFC/sgCC, as well as between DMPFC and medial prefrontal cortex encompassing VMPFC and cingulate gyrus was associated to a better response to rTMS, as demonstrated by decreased HAM-D scores. Furthermore, lower baseline rs-FC between DMPFC and right thalamus, right putamen, right hippocampus/amygdala, as well as between sgACC and putamen, insula, and parahippocampus/amygdala, was associated with better treatment outcomes, measured with HAM-D score [16].

Baseline fMRI findings of a case series study on nine depressed BD patients revealed that the betweenness centrality (BC) was higher in the ventral striatum, right amygdala, DMPFC, temporal pole, a region in left DLPFC, and anterior insula among responders to rTMS. Conversely, non-responders showed significantly higher BC in the left VMPFC, left DLPFC, DMPFC, dorsal anterior cingulate cortex (ACC), right anterior insula, and retrosplenial cingulate cortex. Moreover, by setting a higher threshold and increasing the acceptance stipulations, only a node in the VMPFC had higher connectivity in non-responders than responders. Additionally, it was shown that responders and non-responders had opposite hemispheric lateralization in the rs-FC of selective brain areas to the left VMPFC seed. In particular, non-responders showed higher rs-FC to left VMPFC from DMPFC, DLPFC, posterior cingulate cortex, frontopolar cortex, and MTG in the right hemisphere. Conversely, they had lower rs-FC to the same left VMPFC seed from the same areas in the left side of the brain, including left DMPFC, DLPFC, inferior parietal lobe, occipital cortex, and caudate nucleus [26].

Using a similar modality approach, a study of rTMS response on twenty-six unipolar and BD patients, two with depressive BD, assessed the alterations across eight resting-state networks, consisting of rFPN, lFPN, anterior default mode (aDMN), posterior default mode (pDMN), SN, CEN, vestibular nuclei (VN), and sensorimotor (SMN) networks, and found no association between antidepressant outcomes (measured by the MADRS) and baseline rs-FC, local connectivity or internetwork connectivity [19].

#### 3.2.2. PET Investigations

Implementation of PET as an rTMS predictor tool is reported in several studies as follows: 

Speer et al., (2009) [28] found that the baseline H152O PET hypoperfusion was related to better response and HAM-D improvement when using 20 Hz rTMS, while one Hz rTMS exacerbated depressed mood. The baseline whole-brain hypermetabolism did not present significant changes, but the regional analysis showed a significant correlation between the degree of antidepressant response, measured with HAM-D, and baseline hyperperfusion degree in the VMPFC, bilateral DLPFC, medial and lateral temporal lobe, thalamus, midbrain regions, including substantia nigra and red nucleus, right amygdala, cerebellum, and some regions in frontal to occipital cortices. 

Similarly, an [18F]-FDG-PET combined with MRI exploratory study was conducted with 11 depressed BD patients to determine if baseline metabolism of the specific brain regions was different between rTMS responders and non-responders. PET findings demonstrated some baseline differences among both responders and non-responders compared to healthy subjects, and in comparing responders and non-responders with each other, they found lower cerebral glucose uptake index (gluMI) of the left orbitofrontal cortex (OFC) and higher gluMI values of the areas encompassing uncinate fasciculus, anterior commissure and left amygdala in non-responders. To increase the accuracy and figure out whether the mentioned lower/higher gluMI values are due to the differences in the volumes of the regions or because of their different metabolism, these PET findings were corrected with MRI; the authors found that lower gluMI of the left ACC, left VLPFC, left OFC, and right DLPFC was due to lower volume in these areas. Gray matter volume was reduced in some regions with low gluMI, such as left OFC in non-responders compared to both responders and healthy subjects and left ACC in non-responders compared to healthy subjects. The authors also observed that OFC gluMI and amygdala gluMI were positively correlated with each other in responders, negatively correlated in non-responders, and had no correlations in healthy subjects, ultimately suggesting that amygdala metabolism and OFC volume might be predictors of rTMS response. Furthermore, in non-responders, there was a negative correlation between the gluMI of the left uncus and left OFC, as well as a positive correlation between the gluMI of the OFC and prefrontal regions encompassing ACC, left VLPFC, and left DLPFC [17].

#### 3.2.3. SPECT Investigations

SPECT studies have shown promising outcomes. Specifically, an open-label trial using 99mTc-ECD SPECT was conducted on nine depressed BD patients to identify whether the characteristics of the baseline brain regions correlated with response to rTMS add-on therapy. Comparing the baseline differences among patients and healthy subjects, the authors showed significant hypoperfusion in the brain regions encompassing the bilateral precentral, left postcentral cortices, bilateral anterior cingulate cortices, left superior temporal cortex, left inferior parietal lobule, left insula, and the bilateral inferior, middle, medial and left superior frontal cortices, among patients. Moreover, responders and non-responders revealed different baseline perfusion patterns, with non-responders showing hypoperfusion of the left uncus/parahippocampal cortex, right thalamus, and left medial and bilateral superior frontal cortices. These regions were negatively correlated with the Beck Depression Inventory (BDI) and the State-Trait Anxiety Inventory (STAI) scores on the 20th day. This pattern of hypoperfusion was not present for the responders [24]. The study by Nahas et al., (2001) [29] found that a higher rise in the brain activity (computed with the rCBF) at the stimulation site (left DLPFC) was inversely correlated with the distance from the coil (scalp) to the outer cortex.

### 3.3. Comparison of Unipolar and Bipolar Depression in Terms of rTMS Treatment Response

The comparison in rTMS response between patients with unipolar and bipolar depression was reported in five of the 12 studies. Of these, only one investigation found differences between the two groups and showed better outcomes in patients with bipolar depression relative to unipolar depression [1]. On the other hand, the other four investigations revealed no differences between unipolar and bipolar subjects. Specifically, an fMRI study found no differences in post-rTMS changes based on the BOLD signal [27]. Likewise, Downar et al., (2014) [26] investigated this comparison with HAM-D improvement post-rTMS and found similar results in both disorders. Similarly, the improvement rate of depression scores did not differ between patients with unipolar and bipolar depression in two other trials [16,17].

### 3.4. Side Effects of rTMS in Bipolar Depression

Three studies assessed side effects related to rTMS. All of these reported no side effects, with only one patient experiencing claustrophobia in the scanner in the study by Li et al., (2004) [27].

## 4. Discussion

This systematic review collected evidence on neuroimaging findings and biomarkers related to response to rTMS in patients with bipolar depression. Overall, the included studies showed that lower volumes of superior frontal and caudal middle frontal regions were predictors of response to rTMS, as well as higher connectivity of emotion regulation and executive control regions, including DMPFC-sgACC and sgACC-DLPFC, and lower connectivity between right DMPFC, right DLPFC, right posterior cingulate cortex, right frontopolar cortex, right MTG and left VMPFC. Increased connectivity in the areas near the coil was evident in fMRI studies after rTMS, and also, increased rCBF was observed in PET and SPECT investigations.

### 4.1. Cortical and Subcortical Findings

The findings of this study did not demonstrate changes in cortical volume following rTMS in the short term; however, some of the reviewed investigations found altered performance and changes in activation after rTMS in several cortical areas. Specifically, activation of prefrontal circuits, right medial frontal lobe, left MFG, right OFC, left MTG, and right prefrontal cortex [27,29], and increased blood perfusion in the prefrontal cortex and cingulate gyrus [23] after rTMS were reported, ultimately suggesting the efficacy of this approach in normalizing brain regions often found altered in BD, both at a functional [32] and structural level [33]. Moreover, deficits in prefrontal regions, which are involved in several functions, including executive control and emotion processing have been consistently observed in BD and they have been considered as the basis of the neuropathophysiology of BD [34,35,36]. Additionally, one reviewed study also reported that lower baseline left OFC thickness coupled with hypometabolism of glucose in the region characterized only non-responder patients [17], in line with a previous study showing an association of hypometabolism in OFC with a decrease in gray matter volume in more severely ill patients [37].

With regards to subcortical regions, hypoperfusion in the right thalamus was reported in non-responders to rTMS [24], and bilateral activation of the thalamus [27] and increased blood supply in this area [23] were observed following rTMS in some of the reviewed studies. The thalamus is the largest subcortical structure that modulates mood states and plays an essential role in BD etiology [38]. Indeed, it has been reported that BD patients who did not take lithium had smaller right thalamus volume than healthy subjects [39]. In addition, it has been shown that the presence of glutamatergic system disturbances and neuronal activity dysregulations is associated with deficits in the limbic–thalamo–cortical circuitry in these patients [40]. Additionally, the hippocampus, putamen, and amygdala are subcortical structures that have been extensively found to be implicated in BD [41]. More in detail, some evidence reported the presence of altered excitatory glutamate neurotransmission and deficits in regulating neuronal activity in the hippocampus [40] as well as putamen hypoactivation [42] and altered amygdala activity [43] in BD patients.

Therefore, overall, the results suggest that rTMS might play a key role in normalizing brain activation in cortical and subcortical structures with a subsequent amelioration of clinical symptomatology, although current TMS machines cannot directly stimulate subcortical structures.

### 4.2. Functional Connectivity Changes

The reviewed studies demonstrated that the rs-FC and the interaction between brain structures changed after rTMS treatment in BD, ultimately suggesting that these regions may be used as BD vulnerability biomarkers and response predictors [44,45]. Specifically, a decrease in sgACC-caudate connectivity [16] and a reduction of SN connectivity [19] following rTMS were examples corroborating this hypothesis. 

In addition to these post-rTMS changes, some reviewed studies have reported that the baseline connectivity between structures and networks can be a predictor of treatment response rate, e.g., non-responders to rTMS had lower baseline connectivity in tegmentum and striatum [26], while responders had lower baseline cortico-thalamic, cortico-striatal, and cortico-limbic connectivity and higher baseline DMPFC–sgACC and sgACC-DLPFC connectivity [16]. In general, previous studies have shown that BD is characterized by connectivity dysfunctions in SN and cortico-limbic and cortico-striatal circuits, which may reflect the emotional and cognitive deficits in BD patients [46]. Similarly, the cortico-thalamic circuit is another emotional brain system found altered in BD that may also explain some of the core symptoms of BD [47]. 

In sum these findings suggest that (a) rTMS seems to normalize functional connectivity across key regions known to be involved in BD and (b) the impaired baseline connectivity between selective brain structures in patients with BD can be a predictor of response to rTMS. 

### 4.3. Comparison between rTMS in Unipolar and Bipolar Depression 

Two studies directly compared the effects of rTMS in BD and MDD and found no significant differences [16,17]. The therapeutic effect of rTMS, primarily proposed for resistant MDD, has been extended to bipolar depression in previous studies [48,49]. The similar efficacy of rTMS in unipolar and bipolar depressed patients suggests that this technique is effective regardless of the underlying disorder. However, a recent investigation has shown that left-sided rTMS resulted in greater changes in the patient health questionnaire (PHQ-9), approximately double the response (80% vs. 39%), and triple the remission (45% vs. 15%) in patients with bipolar depression compared to patients with unipolar depression. In this study, unilateral treatment protocol, non-lithium mood stabilizers, male gender, and the number of treatments were predictors of PHQ-9 improvement [50].

### 4.4. Strengths and Limitations

This review provides a comprehensive overview of the neuroimaging predictors of rTMS response in patients with bipolar depression. A systematic literature search was conducted on four databases, with a predefined search strategy and no language restrictions. Keywords and a detailed list of excluded studies have been provided to guarantee replicability and transparency. However, it also has some limitations that need to be mentioned. First, the included studies were heterogeneous, especially in terms of the type of TMS protocol, imaging modality, and study type, e.g., case series, cohort study, open-label trial, and randomized controlled trial. Second, a narrative synthesis of high-quality studies was not performed separately. Third, the majority of the reviewed studies included a sample of patients with either unipolar or bipolar depression diagnoses. However, the studies that directly compared the two patient groups did not find any significant difference and we can therefore indirectly assume that TMS treatment may have the same effect on the two groups of subjects. Finally, the included studies also had limitations that affect the conclusion, including small study size, short duration of follow-up (low frequency of imaging points per patient), and the presence of concurrent treatments in patients.

### 4.5. Clinical Implications

TMS can provide valuable insights into the underlying biological mechanisms of BD and the patient’s response to treatment when combined with neuroimaging modalities. One of the significant clinical implications of using neuroimaging methods with TMS is their ability to predict the clinical response to treatment. By analyzing brain activity and structural changes before and after TMS therapy, clinicians could determine which patients are more likely to respond positively to the treatment. This personalized approach to treatment can lead to improved patient outcomes, reduced healthcare costs, and increased patient satisfaction. Furthermore, neuroimaging techniques can be used to monitor the effects of TMS therapy and adjust the treatment plan as needed. By tracking alterations in brain activity and structure, clinicians can assess the effectiveness of the treatment and make adjustments to optimize patient outcomes. This study reviewed significant clinical implications of neuroimaging modalities for prognosis, treatment planning, and patient outcomes in patients with bipolar depression who received TMS.

## 5. Conclusions

The findings of the reviewed studies support the effects of rTMS on brain activation, functional connectivity, and brain metabolism of cortical and subcortical brain areas in BD; several of these effects correlated with response to rTMS. 

The main neuroimaging correlates of rTMS response observed in the reviewed studies included (i) right insula activation; (ii) changes in the activity of prefrontal and subcortical circuits in concert with ventromedial prefrontal deactivation; (iii) increase in dmPFC-thalamus connectivity; (iv) decrease in the connectivity of sgACC-caudate, dmPFC-insula, sgACC-midcingulate cortex, and dmPFC and parahippocampal gyrus/amygdala; and (v) reduced SN connectivity. 

Moreover, several baseline neuroimaging findings were correlated with response to rTMS, including (i) hyperperfusion of cerebellum, amygdala, and other midbrain and cortical areas (before 1 Hz rTMS); (ii) hypoperfusion in PET scan (before 20 Hz rTMS); (iii) rCBF of frontal and temporal areas; (iv) hypometabolism of temporal-limbic regions, especially amygdala; (v) higher GM in OFC; (vi) lower GM in anterior commissure and left uncinated fasciculus; (vii) connectivity of dmPFC and sgACC with each other and with other cortical, thalamic, and striatal regions; (viii) higher connectivity of dmPFC-sgACC and sgACC-dlPFC; and (ix) lower connectivity of cortico-thalamic (dmPFC-medial dorsal thalamus), cortico-striatal (dmPFC-putamen), and cortico-limbic (sgACC-amygdala and sgACC-hippocampus).

As a treatment option for resistant depressed patients with BD, rTMS pursues the target of pharmacotherapy and psychotherapy but with a different mechanism [51]. Psychological and pharmacological interventions cause indirect reorganization in neural circuits, while brain stimulation interventions directly modify the activity of these circuits, which underlies the importance of neuroanatomical predictors, including grey matter volume, in stimulation interventions [1]. Indeed, imaging modalities are used to precisely target areas and networks by stimulation interventions, to predict treatment response, and to evaluate treatment response. In addition to these remarkable advantages, imaging modalities also have significant disadvantages. The need to access the modality, specialized data processing, and the high cost are some of their limitations. Demographic and clinical markers can be used as alternatives, although the role of neuroimaging findings seems to be more prominent [18]. 

The results reinforce the need to identify neuroimaging and clinical biomarkers that can predict the response to treatment. Still, attention remains in this area to understand how to optimize rTMS for each patient to achieve a better response to treatment. Recording of imaging data from different modalities at multiple time points after rTMS is recommended in further studies.

## Figures and Tables

**Table 1:** The characteristics of the included studies.

Study, Year	Study Type	Participants	TMS Responders and Non-Responders	Intervention	Imaging Modality	Brain Networks	Baseline Findings	Baseline Correlations	Post-Treatment Findings	Post-Treatment Correlations
Speer et al., 2000 [23]	Double-blind, placebo-controlled, crossover study	N = 8 MDD N = 2 BD (depressive phase)	NR	10 Hz rTMS / 1 Hz rTMS 2 weeks, 10 daily or two groups of 5 daily	PET	NR	NR	NR	1. An amount of 20 HZ rTMS: ↑ rCBF in the cingulate gyrus (L >> R), prefrontal cortex (L > R), left amygdala, uncus, basal ganglia, bilateral insula, hippocampus, parahippocampus, cerebellum and thalamus. 2. An amount of 1 Hz rTMS: ↓ rCBF in the right prefrontal cortex, left medial temporal cortex, left basalganglia, and left amygdala 3. gCBF ↑ after 20 Hz rTMS	1. Negative correlation between the HAM-D change in the same patients after 10 Hz vs. 1 Hz rTMS and vice versa. 2. No correlation between blood flow change and clinical response
Nahas et al., 2001 [29]	Double-blind placebo- controlled trial	N = 16 MDD N = 7 BD (depressive phase) 9 received 20 Hz, 5 received 5 Hz and 9 were placebo group	Responders = 7 Non responders = 16	rTMS (5 and 20 Hz) 10 sessions over 2 weeks	(ECD) SPECT + MRI (T1 weighted 3D)	NR	NR	NR	1. Active rTMS changes compared to baseline: ↑ activity of right medial frontal lobe and left middle frontal gyrus, ↓ activity of left anterior cingulate, anterior temporal, left uncus and left insula 2. Active rTMS group compared to the placebo group: lower activation of the bilateral parietal and right thalamus 3. Fast stimulation: ↑ in activity directly below the coil, ↓ of activity in midcingulate gyrus and left paralimbic area (versus slow stimulation) 4. Active rTMS group compared to baseline relative to the placebo: ↓ activity of the left middle temporal gyrus	1. The improvement and number of responders: active rTMS group >> placebo group 2. No significant differences in the rCBF changes after the fast and slow stimulation 3. Negative correlation between the distance from stimulation site, and the blood flow
Li et al., 2004 [27]	Trial	N = 6 MDD N = 8 BD (depressive phase)	NR	rTMS 147 stimuli for each subject, 1 Hz	fMRI	NR	NR	NR	1. Post-rTMS significant activation in the following regions (compared with baseline): ipsilateral hippocampus, bilateral thalamus (ipsilateral mediodorsal, bilateral pulnivar, anterior nucleus), bilateral putamen, bilateral parietal lobes and insula, right orbitofrontal cortex, left middle temporal cortex, and right prefrontal cortex 2. Deactivation in the right ventromedial prefrontal cortex during the rTMS	1. Positive correlation between the right insula activation and HAM-D score 2. No correlation between changes in the brain regions after TMS and age or motor threshold 3. Change in the activity of prefrontal and subcortical circuits post-rTMS (both changes in concert with ventromedial prefrontal deactivation)
Speer et al., 2009 [28]	Double-blind, sham-controlled, cross-over	N = 13 MDD N = 9 BD (depressive phase)	NR	20 Hz or 1 Hz rTMS/ sham rTMS 10 daily sessions, 5 days per week	FDG-PET/H^15^_2_O PET + MRI	NR	NR	1. Correlation between the antidepressant response following 1 Hz rTMS and the baseline hyperperfusion of following brain regions: the cerebellum, amygdala, other midbrain and cortical areas. 2. Association between the hypoperfusion in baseline PET scan and better response of 20 Hz rTMS	1. Improvements in one frequency resulting in worse outcomes in the other frequency (among 19 patients receiving both)	1. No correlation between clinical and demographic characteristics and response to either 1 or 20 Hz
Richieri et al., 2011 [24]	Open label trial	N = 24 MDD N = 9 BD (depressive phase)	Responders = 18 (7 BD) Non responders = 15 (2 BD)	rTMS 4 weeks, 5 sessions each week, 20 sessions, 10 Hz	ECD SPECT	NR	1. Hypoperfusion in the following regions in patients compared to healthy group: frontal and temporal (areas encompassing bilateral anterior cingulate, left post central and bilateral precentral cortices, left insula, left superior temporal, left inferior parietal, and bilateral inferior, medial, middle, and left superior frontal cortices) (left-side dominance) 2. Hypoperfusion in the following regions in non-responders: the left medial and bilateral superior frontal, left medial temporal, left uncus /parahippocampal cortex and right thalamus 3. No hypoperfusion in responders compared to non-responders	1. Strong correlation between pretreatment rCBF of the mentioned brain regions and treatment response 2. Negative correlation between the hypoperfusion and BDI scores	NR	1. No association between clinical/demographic characteristics and rTMS response
Martinot et al., 2011 [17]	Double-blind, randomized, sham-controlled trial	N = 20 MDD N = 11 BD (depressive phase; 8 BD-1 and 3 BD-2) N = 39 HC	Responders = 17 (6 BD) Non responders = 14 (5 BD)	rTMS 10 sessions, 10 Hz	MRI + [18F]-FDG-PET	NR	1. No difference in two groups 2. Non responders: ↓ prefrontal metabolism (mostly in the insula, OFC, and ACC) and ↑ temporal-limbic metabolism 3. Non-responders: ↓ GM volume in rostral part of left OFC and left ACC	1. A possible correlation between hypermetabolism of the temporal-limbic regions (especially amygdala) and nonresponse to rTMS 2. A possible correlation between the GM reduction in OFC and negative response to rTMS 3. Changes in white matter tracts of ventral frontal-temporal-limbic network (higher glucose metabolism in anterior commissure and left uncinated fasciculus) being a possible cause of resistance to rTMS	NR	1. No difference between BD and MDD regarding the improvement in depression scores rate
Downar et al., 2013 [26]	Open label design case series	N = 38 MDD N = 9 BD (depressive phase; 2 BD-1 and 7 BD-2)	Responders = 24 Non responders = 23	rTMS 20 sessions, 10 Hz; those with response but not remission had 10 additional sessions in 2 weeks	fMRI	NR	1. Responders: ↑ BC in right amygdala, ventral striatum, temporal pole, and DMPFC, besides a region in left DLPFC and anterior insula. 2. Non-responders: ↑ BC in left VMPFC, regions in left DLPFC, DMPFC, dorsal ACC, retrosplenial cingulate cortex, and right anterior insula 3. Non-responders: ↓ connectivity in tegmental area, striatum, and a region in ventromedial prefrontal cortex. 4. Non-responders: ↑ baseline anhedonia symptoms 5. Different hemispheric lateralization between two groups (dorsomedial/dorsolateral regions to ventromedial region connectivity)	NR	1. Post-rTMS HAM-D score changes: ≥ 50% symptom ↓ in 51.1% and remission in 42.6% 2. Post-rTMS BDI-II score changes: ≥ 50% symptom ↓ in 48.9% and remission in 44.7%	NR
Solomons et al., 2014 [16]	Open-label trial	N = 21 MDD N = 4 BD (depressive phase; 1 BD-1 and 3 BD-2)	NR	DmPFC rTMS 4 weeks, 5 sessions per week, 20 sessions, 10 Hz	rs-fMRI	Two main seeds: dmPFC, sgACC	NR	1. Correlation between better rTMS response and the baseline connectivity of dmPFC and sgACC with each other and with other cortical, thalamic, and striatal regions 2. Correlation between better response and the ↑ baseline connectivity of dmPFC –sgACC and sgACC- dlPFC 3. Correlation between better response and ↓ connectivity of cortico-thalamic (dmPFC-medial dorsal thalamus), cortico-striatal (dmPFC-putamen), cortico-limbic (sgACC-amygdala and sgACC-hippocampus)	1. A 45% ↓ in HAM-D score post-rTMS treatment 2.higher baseline and more post-rTMS ↓ in HAM-D score in BD compared to MDD (however not significant)	1. Correlation between rTMS response improvement and changes from baseline to post-rTMS in the following regions: ↑ in dmPFC-thalamus connectivity and ↓ in the connectivity of sgACC-caudate; dmPFC-insula, sgACC-midcingulate cortex, and dmPFC and parahippocampal gyrus/amygdala 2. No difference among BD and MDD patients regarding the outcomes (no obvious change occurred in results by excluding the four BD patients from the study)
Diederichs et al., 2021 [22]	Double-blind randomized controlled study	N = 7 sham-iTBS, N = 10 active-iTBS The paired pre-post data were available for: N = 6 sham iTBS N = 6 active iTBS All were acute BD depressive phase (6 BD-1 and 12 BD-2)	NR	iTBS 600 pulses each session, delivered as triplets of 50 Hz repeated at 5 Hz	GABA edited MRS + MRI (T1 weighted to find the participants’ left DLPFC at baseline)	Medial prefrontal cortex (mPFC)	No GABA level difference between two groups, but higher Glx in the sham group	1. No association between GABA and clinical characteristics (except for HAM-D score) 2. No association between Glx and clinical characteristics	Active iTBS group: ↑ in mPFC GABA	1. No change in Glx. 2. No correlation between the GABA effect and antidepressant effect or anhedonia
Godfrey et al., 2022 [19]	Open label naturalistic study	N = 24 MDD N = 2 BD (depressive phase)	Responders = 11 Non-responders = 15 All BD were nonresponders	rTMS weekday treatment for 4 weeks, 20 sessions, 10 Hz	fMRI	8 RSNs: SN, aDM, pDMN, rFPN, lFPN, CEN, VN, SMN	NR	1. No correlation between the antidepressant response and baseline connectivity of the eight RSNs. 2. No correlation between baseline fALFF and Δ MADRS	NR	1. ↓ SN connectivity post rTMS (correlated with Δ MADRS) 2. ↑ Internetwork connectivity between pDMN and SN (not correlated with antidepressant response) 3. ↑ fALFF in pDMN, SN, CEN, lFPN, and rFPN (not correlated with Δ MADRS)
Harika-Germaneau et al., 2022 [1]	Retrospective naturalistic cohort study	N = 71 MDD N = 24 BD (depressive phase)	Responders = 38 Non-responders = 57 among BD patients, 14 were responders and 10 were nonresponders	rTMS 5 times a week, for two weeks, 10 sessions, 20 Hz	MRI (T1)	NR	NR	Better response to rTMS in women, BD patients and those who were less resistant to rTMS	1. Responders: a ↓ in the volume of the left hemisphere in superior frontal and caudal middle frontal regions (not in right hemisphere)	1. No significance difference in the cortical thickness between responders and non-responders

ACC: anterior cingulate cortex; aDMN: anterior default mode network; BC: betweenness centrality; BD: bipolar disorder; BD-1: bipolar disorder type 1; BD-2: bipolar disorder type 2; BDI-II: Beck’s depression inventory; CEN: central executive network; DLPFC: dorsolateral prefrontal cortex; DMPFC: dorsomedial prefrontal cortex; ECD: 99mTc-ethyl cysteinate dimer; fALFF: fractional amplitude of low-frequency fluctuations; [18F]-FDG-PET:18F-fluorodeoxyglucose positron emission tomography; fMRI: functional magnetic resonance imaging; GABA: gamma-aminobutyric acid; gCBF: global cerebral blood flow; Glx: glutamate + glutamine; GM: gray matter; HAM-D: Hamilton Rating Scale for Depression; HC: healthy comparison; iTBS: intermittent theta-burst stimulation; L: left; lFPN: left frontoparietal network; MADRS: Montgomery-Åsberg Depression Rating Scale; MDD: major depressive disorder; MRI: magnetic resonance imaging; MRS: Magnetic resonance spectroscopy; NR: not reported; OFC: orbitofrontal cortex; pDMN: posterior default mode network; PET: positron emission tomography; R: right; rCBF: regional cerebral blood flow; rFPN: right frontoparietal network; rs-fMRI: resting state fMRI; RSNs: resting state networks; rTMS: repetitive transcranial magnetic stimulation; sgACC: the subgenual region of the ACC; SMN: sensorimotor network; SN: salience network; SPECT: single photon emission computed tomography; VMPFC: ventromedial prefrontal cortex; VN: visual. ↑: increased; ↓: decreased.

## Data Availability

No new data were created or analyzed in this study. Data sharing is not applicable to this article.

## Supplementary Materials

The following supporting information can be downloaded at: https://www.mdpi.com/article/10.3390/brainsci13050801/s1.

## Appendix A. The Searched Terms in ISI Web of Science Core Collection, Embase, and Medline and the Number of Obtained Results

**Table A1:** Databases, strings searched in them, and the total number of obtained citations.

Database	Terms	Results
Web of Science	(TS=(bipolar) OR TS=(mania) OR TS=(manic) OR TS=(hypomani*)) AND (“transcranial magnetic stimulation” (Topic) or “theta burst” (Topic) or “repetitive transcranial” (Topic) or TMS (Topic) or rTMS (Topic)) AND (TS=(“functional MRI”) OR TS=(fMRI) OR TS=(MRI) OR TS=(“Magnetic resonance”) OR TS=(tomography) OR TS=(SPECT) OR TS=(neuroimaging) OR TS=(“functional connectivity”) OR TS=(“neural network activity”) OR TS=(“regional homogeneity”) OR TS=(DTI) OR TS=(“diffusion tensor imaging”) OR TS=(PET) OR TS=(imaging))	125
Embase	(bipolar:ti,ab,kw OR mania:ti,ab,kw OR manic:ti,ab,kw OR hypomani*:ti,ab,kw) AND (‘transcranial magnetic stimulation’:ti,ab,kw OR ‘theta burst’:ti,ab,kw OR ‘repetitive transcranial’:ti,ab,kw OR tms:ti,ab,kw OR rtms:ti,ab,kw) AND (‘functional mri’:ti,ab,kw OR fmri:ti,ab,kw OR mri:ti,ab,kw OR ‘magnetic resonance’:ti,ab,kw OR tomography:ti,ab,kw OR spect:ti,ab,kw OR neuroimaging:ti,ab,kw OR ‘functional connectivity’:ti,ab,kw OR ‘neural network activity’:ti,ab,kw OR ‘regional homogeneity’:ti,ab,kw OR dti:ti,ab,kw OR ‘diffusion tensor imaging’:ti,ab,kw OR pet:ti,ab,kw OR imaging:ti,ab,kw)	95
Medline	(TS=(bipolar) OR TS=(mania) OR TS=(manic) OR TS=(hypomani*)) AND (“transcranial magnetic stimulation” (Topic) or “theta burst” (Topic) or “repetitive transcranial” (Topic) or TMS (Topic) or rTMS (Topic)) AND (TS=(“functional MRI”) OR TS=(fMRI) OR TS=(MRI) OR TS=(“Magnetic resonance”) OR TS=(tomography) OR TS=(SPECT) OR TS=(neuroimaging) OR TS=(“functional connectivity”) OR TS=(“neural network activity”) OR TS=(“regional homogeneity”) OR TS=(DTI) OR TS=(“diffusion tensor imaging”) OR TS=(PET) OR TS=(imaging))	51
