# Supplementary material for: Neuroimaging Correlates of Treatment Response to Transcranial Magnetic Stimulation in Bipolar Depression: A Systematic Review
Source: Brain Sci. 2023 May 15;13(5):801. doi: 10.3390/brainsci13050801 (PMC10216729; doi:10.3390/brainsci13050801)
Supplement: Supplementary file 1 [file brainsci-13-00801-s001.zip › brainsci-2371572-supplementary.pdf]

Appendix S1. The Reasons for Exclusion of Full-Texts Assessed for Eligibility

- **No participants of interest**

Maier, M.A.; Armand, J.; Kirkwood, P.A.; Yang, H.W.; Davis, J.N.; Lemon, R.N. Differences in the corticospinal projection from primary motor cortex and supplementary motor area to macaque upper limb motoneurons: An anatomical and electrophysiological study. Cereb. Cortex 2002, 12, 281–296, doi:10.1093/cercor/12.3.281 .

Wilson, T.W.; Slason, E.; Hernandez, O.O.; Asherin, R.; Reite, M.L.; Teale, P.D.; Rojas, D.C. Aberrant high-frequency desynchronization of cerebellar cortices in early-onset psychosis. Psychiatry Res. Neuroimaging 2009, 174, 47–56, doi:10.1016/j.pscychresns.2009.03.009.

Iwabuchi, S.J.; Auer, D.P.; Raschke, F.; Lankappa, S.; Palaniyappan, L. Alterations in the effective connectivity of the fronto-insular network following targeted thetaburst transcranial magnetic stimulation. Bipolar Disord. 2015, 17, 83, doi:10.1111/bdi.12309.

Li, X.B.; Large, C.H.; Ricci, R.; Taylor, J.J.; Nahas, Z.; Bohning, D.E.; Morgan, P.; George, M.S. Using interleaved transcranial magnetic stimulation/functional magnetic resonance imaging (fMRI) and dynamic causal modeling to understand the discrete circuit specific changes of medications: Lamotrigine and valproic acid changes in motor or prefrontal effective connectivity. Psychiatry Res. Neuroimaging 2011, 194, 141–148, doi:10.1016/j.pscychresns.2011.04.012.

Hayasaka, S.; Nakamura, M.; Noda, Y.; Izuno, T.; Saeki, T.; Iwanari, H.; Hirayasu, Y. Lateralized hippocampal volume increase following high-frequency left prefrontal repetitive transcranial magnetic stimulation in patients with major depression. Psychiatry Clin. Neurosci. 2017, 71, 747–758, doi:10.1111/pcn.12547.

Li, X.B.; Teneback, C.C.; Nahas, Z.; Kozel, F.A.; Large, C.; Cohn, J.; Bohning, D.E.; George, M.S. Interleaved transcranial magnetic stimulation/functional MRI confirms that lamotrigine inhibits cortical excitability in healthy young men. Neuropsychopharmacology 2004, 29, 1395–1407, doi:10.1038/sj.npp.1300452.

Wang, Y.; Liu, C.; Fang, T.; Lin, X.; Jiang, D.; Zhu, J.; Zhuo, C.; Ye, J. Cognitive Improvements from Adjunct Therapy with Transcranial Magnetic Stimulation are Short-Lived In Patients with Remitted Bipolar Disorder. Psychiatry and Clinical Psychopharmacology 2020, 30, 346–353, doi:10.5455/PCP.20200221124442.

- **No intervention of interest**

Lan, M.J.; Chhetry, B.T.; Oquendo, M.A.; Sublette, M.E.; Sullivan, G.; Mann, J.J.; Parsey, R.V. Cortical thickness differences between bipolar depression and major depressive disorder. Bipolar Disord. 2014, 16, 378–388, doi:10.1111/bdi.12175.

Rai, S.; Griffiths, K.R.; Breukelaar, I.A.; Barreiros, A.R.; Chen, W.T.; Boyce, P.; Hazell, P.; Foster, S.L.; Malhi, G.S.; Harris, A.W.F.; et al. Default-mode and fronto-parietal network connectivity during rest distinguishes asymptomatic patients with bipolar disorder and major depressive disorder. Transl. Psychiatr. 2021, 11, 8, doi:10.1038/s41398-021-01660-9 .

Eryilmaz, G.; Onen Unsalver, B.; Gogcegoz, G.; Saglam, E. Demographic evaluation of patients treated with transcranial direct current stimulation (TDCS) in a university hospital. European Neuropsychopharmacology 2013, 23, S242–S243, doi:10.1016/S0924-977X(13)70376-6 .

Brady, R.O.; Tandon, N.; Masters, G.A.; Margolis, A.; Cohen, B.M.; Keshavan, M.; Ongur, D. Differential brain network activity across mood states in bipolar disorder. J. Affect. Disord. 2017, 207, 367–376, doi:10.1016/j.jad.2016.09.041.

Leow, A.; Ajilore, O.; Zhan, L.; Arienzo, D.; GadElkarim, J.; Zhang, A.F.; Moody, T.; Van Horn, J.; Feusner, J.; Kumar, A.; et al. Impaired Inter-Hemispheric Integration in Bipolar Disorder Revealed with Brain Network Analyses. Biol. Psychiatry 2013, 73, 183–193, doi:10.1016/j.biopsych.2012.09.014.

Rohan, M.; Parow, A.; Stoll, A.L.; Demopulos, C.; Friedman, S.; Dager, S.; Hennen, J.; Cohen, B.M.; Renshaw, P.F. Low-field magnetic stimulation in bipolar depression using an MRI-based stimulator. Am. J. Psychiat. 2004, 161, 93–98, doi:10.1176/appi.ajp.161.1.93.

Nahas, Z.; Teneback, C.; Chae, J.H.; Mu, Q.W.; Molnar, C.; Kozel, F.A.; Walker, J.; Anderson, B.; Koola, J.; Kose, S.; et al. Serial vagus nerve stimulation functional MRI in treatment-resistant depression. Neuropsychopharmacology 2007, 32, 1649–1660, doi:10.1038/sj.npp.1301288.

Tso, I.; Angstadt, M.; Johnson, T.; Diwadkar, V.; Taylor, S. Visual disturbances underlie abnormal eye gaze perception in psychosis: Psychophysical and effective connectivity evidence. Schizophr. Bull. 2019, 45, S114–S115, doi:10.1093/schbul/sbz022.063.

Marten, L.E.; Singh, A.; Muellen, A.M.; Noack, S.M.; Kozyrev, V.; Schweizer, R.; Goya-Maldonado, R. Altered functional connectivity relates to motor performance deficits in bipolar but not unipolar depression. 2021, doi:10.1101/2021.11.26.21266905.

Brady, R.; Beermann, A.; Nawaz, U.; Keshavan, M.; Ongur, D.; Halko, M.; Lewandowski, K. Cerebellar disconnectivity impairs information processing speed in bipolar disorder and schizophrenia. Bipolar Disord. 2020, 22, 37, doi:10.1111/bdi.12934.

Anticevic, A.; Cole, M.W.; Repovs, G.; Murray, J.D.; Brumbaugh, M.S.; Winkler, A.M.; Savic, A.; Krystal, J.H.; Pearlson, G.D.; Glahn, D.C. Characterizing Thalamo-Cortical Disturbances in Schizophrenia and Bipolar Illness. Cereb. Cortex 2014, 24, 3116–3130, doi:10.1093/cercor/bht165.

Liberg, B.; Klauser, P.; Harding, I.H.; Adler, M.; Rahm, C.; Lundberg, J.; Masterman, T.; Wachtler, C.; Jonsson, T.; Kristoffersen-Wiberg, M.; et al. Functional and structural alterations in the cingulate motor area relate to decreased fronto-striatal coupling in major depressive disorder with psychomotor disturbances. Front. Psychiatry 2014, 5, 9, doi:10.3389/fpsyt.2014.00176.

- **No outcome of interest**

Lan, M.J.; Chhetry, B.T.; Oquendo, M.A.; Sublette, M.E.; Sullivan, G.; Mann, J.J.; Parsey, R.V. Cortical thickness differences between bipolar depression and major depressive disorder. Bipolar Disord. 2014, 16, 378–388, doi:10.1111/bdi.12175.

Rai, S.; Griffiths, K.R.; Breukelaar, I.A.; Barreiros, A.R.; Chen, W.T.; Boyce, P.; Hazell, P.; Foster, S.L.; Malhi, G.S.; Harris, A.W.F.; et al. Default-mode and fronto-parietal network connectivity during rest distinguishes asymptomatic patients with bipolar disorder and major depressive disorder. Transl. Psychiatr. 2021, 11, 8, doi:10.1038/s41398-021-01660-9 .

Eryilmaz, G.; Onen Unsalver, B.; Gogcegoz, G.; Saglam, E. Demographic evaluation of patients treated with transcranial direct current stimulation (TDCS) in a university hospital. European Neuropsychopharmacology 2013, 23, S242–S243, doi:10.1016/S0924-977X(13)70376-6 .

Brady, R.O.; Tandon, N.; Masters, G.A.; Margolis, A.; Cohen, B.M.; Keshavan, M.; Ongur, D. Differential brain network activity across mood states in bipolar disorder. J. Affect. Disord. 2017, 207, 367–376, doi:10.1016/j.jad.2016.09.041.

Leow, A.; Ajilore, O.; Zhan, L.; Arienzo, D.; GadElkarim, J.; Zhang, A.F.; Moody, T.; Van Horn, J.; Feusner, J.; Kumar, A.; et al. Impaired Inter-Hemispheric Integration in Bipolar Disorder Revealed with Brain Network Analyses. Biol. Psychiatry 2013, 73, 183–193, doi:10.1016/j.biopsych.2012.09.014.

Rohan, M.; Parow, A.; Stoll, A.L.; Demopulos, C.; Friedman, S.; Dager, S.; Hennen, J.; Cohen, B.M.; Renshaw, P.F. Low-field magnetic stimulation in bipolar depression using an MRI-based stimulator. Am. J. Psychiat. 2004, 161, 93–98, doi:10.1176/appi.ajp.161.1.93.

Nahas, Z.; Teneback, C.; Chae, J.H.; Mu, Q.W.; Molnar, C.; Kozel, F.A.; Walker, J.; Anderson, B.; Koola, J.; Kose, S.; et al. Serial vagus nerve stimulation functional MRI in treatment-resistant depression. Neuropsychopharmacology 2007, 32, 1649–1660, doi:10.1038/sj.npp.1301288.

Tso, I.; Angstadt, M.; Johnson, T.; Diwadkar, V.; Taylor, S. Visual disturbances underlie abnormal eye gaze perception in psychosis: Psychophysical and effective connectivity evidence. Schizophr. Bull. 2019, 45, S114–S115, doi:10.1093/schbul/sbz022.063.

Marten, L.E.; Singh, A.; Muellen, A.M.; Noack, S.M.; Kozyrev, V.; Schweizer, R.; Goya-Maldonado, R. Altered functional connectivity relates to motor performance deficits in bipolar but not unipolar depression. 2021, doi:10.1101/2021.11.26.21266905.

Brady, R.; Beermann, A.; Nawaz, U.; Keshavan, M.; Ongur, D.; Halko, M.; Lewandowski, K. Cerebellar disconnectivity impairs information processing speed in bipolar disorder and schizophrenia. Bipolar Disord. 2020, 22, 37, doi:10.1111/bdi.12934.

Anticevic, A.; Cole, M.W.; Repovs, G.; Murray, J.D.; Brumbaugh, M.S.; Winkler, A.M.; Savic, A.; Krystal, J.H.; Pearlson, G.D.; Glahn, D.C. Characterizing Thalamo-Cortical Disturbances in Schizophrenia and Bipolar Illness. Cereb. Cortex 2014, 24, 3116–3130, doi:10.1093/cercor/bht165.

Liberg, B.; Klauser, P.; Harding, I.H.; Adler, M.; Rahm, C.; Lundberg, J.; Masterman, T.; Wachtler, C.; Jonsson, T.; Kristoffersen-Wiberg, M.; et al. Functional and structural alterations in the cingulate motor area relate to decreased fronto-striatal coupling in major depressive disorder with psychomotor disturbances. Front. Psychiatry 2014, 5, 9, doi:10.3389/fpsyt.2014.00176.

Zeeuws, D.; Baeken, C. Intensive HF-rTMS treatment in affective disorders: Sample cases. Eur. Psychiat. 2011, 26, doi:10.1016/S0924-9338(11)72865-6.

George, M.S.; Speer, A.M.; Molloy, M.; Nahas, Z.; Teneback, C.C.; Risch, S.C.; Arana, G.W.; Ballenger, J.C.; Post, R.M. Low frequency daily left prefrontal rTMS improves mood in bipolar depression: A placebo-controlled case report. Hum. Psychopharmacol.-Clin. Exp. 1998, 13, 271-275.

Martis, B.; Alam, D.; Dowd, S.M.; Hill, S.K.; Sharma, R.P.; Rosen, C.; Pliskin, N.; Martin, E.; Carson, V.; Janicak, P.G. Neurocognitive effects of repetitive transcranial magnetic stimulation in severe major depression. Clin. Neurophysiol. 2003, 114, 1125–1132, doi:10.1016/s1388-2457(03)00046-4 .

Novák, T.; Kostýlková, L.; Bareš, M.; Voráčková, V.; Hejzlar, M.; Renka, J.; Baumann, S.; Klírová, M. Repetitive transcranial magnetic stimulation (rTMS) in the treatment of bipolar depression. Brain Stimul. 2021, 14, 1589, doi:10.1016/j.brs.2021.10.004.

Bakker, N.; Shahab, S.; Giacobbe, P.; Blumberger, D.M.; Daskalakis, Z.J.; Kennedy, S.H.; Downar, J. rTMS of the Dorsomedial Prefrontal Cortex for Major Depression: Safety, Tolerability, Effectiveness, and Outcome Predictors for 10 Hz Versus Intermittent Theta-burst Stimulation. Brain Stimul. 2015, 8, 208–215, doi:10.1016/j.brs.2014.11.002.

Hadley, D.; Anderson, B.S.; Borckardt, J.J.; Arana, A.; Li, X.B.; Nahas, Z.; George, M.S. Safety, Tolerability, and Effectiveness of High Doses of Adjunctive Daily Left Prefrontal Repetitive Transcranial Magnetic Stimulation for Treatment-Resistant Depression in a Clinical Setting. J. Ect 2011, 27, 18–25, doi:10.1097/YCT.0b013e3181ce1a8c .

Myczkowski, M.L.; Fernandes, A.; Moreno, M.; Valiengo, L.; Lafer, B.; Moreno, R.A.; Padberg, F.; Gattaz, W.; Brunoni, A.R. Cognitive outcomes of TMS treatment in bipolar depression: Safety data from a randomized controlled trial. J. Affect. Disord. 2018, 235, 20–26, doi:10.1016/j.jad.2018.04.022.

Altamura, A.C.; Dell'Osso, B.; Mariotti, M. Transcranial Magnetic Stimulation (TMS) combined with navigated brain stimulation in drug-resistant bipolar depression: A case report. Italian Journal of Psychopathology 2006, 12, 444–446.

Andrews, S.C.; Enticott, P.G.; Hoy, K.E.; Thomson, R.H.; Fitzgerald, P.B. Reduced mu suppression and altered motor resonance in euthymic bipolar disorder: Evidence for a dysfunctional mirror system? Soc. Neurosci. 2016, 11, 60–71, doi:10.1080/17470919.2015.1029140.

Basavaraju, R.; Mehta, U.M.; Pascual-Leone, A.; Thirthalli, J. Elevated mirror neuron system activity in bipolar mania: Evidence from a transcranial magnetic stimulation study. Bipolar Disord. 2019, 21, 259–269, doi:10.1111/bdi.12723.

Basavaraju, R.; Sanjay, T.N.; Mehta, U.M.; Muralidharan, K.; Thirthalli, J. Cortical inhibition in symptomatic and remitted mania compared to healthy subjects: A cross-sectional study. Bipolar Disord. 2017, 19, 698–703, doi:10.1111/bdi.12546.

Canali, P.; Casarotto, S.; Rosanova, M.; Sferrazza-Papa, G.; Casali, A.G.; Gosseries, O.; Massimini, M.; Smeraldi, E.; Colombo, C.; Benedetti, F. Abnormal brain oscillations persist after recovery from bipolar depression. European psychiatry: the journal of the Association of European Psychiatrists 2017, 41, 10–15, doi:10.1016/j.eurpsy.2016.10.005 .

Rossini, D.; Lucca, A.; Magri, L.; Malaguti, A.; Smeraldi, E.; Colombo, C.; Zanardi, R. A Symptom-Specific Analysis of the Effect of High-Frequency Left or Low-Frequency Right Transcranial Magnetic Stimulation over the Dorsolateral Prefrontal Cortex in Major Depression. Neuropsychobiology 2010, 62, 91–97, doi:10.1159/000315439.

Kazemi, R.; Rostami, R.; Khomami, S.; Baghdadi, G.; Rezaei, M.; Hata, M.; Aoki, Y.; Ishii, R.; Iwase, M.; Fitzgerald, P.B. Bilateral Transcranial Magnetic Stimulation on DLPFC Changes Resting State Networks and Cognitive Function in Patients With Bipolar Depression. Front. Hum. Neurosci. 2018, 12, 10, doi:10.3389/fnhum.2018.00356.
